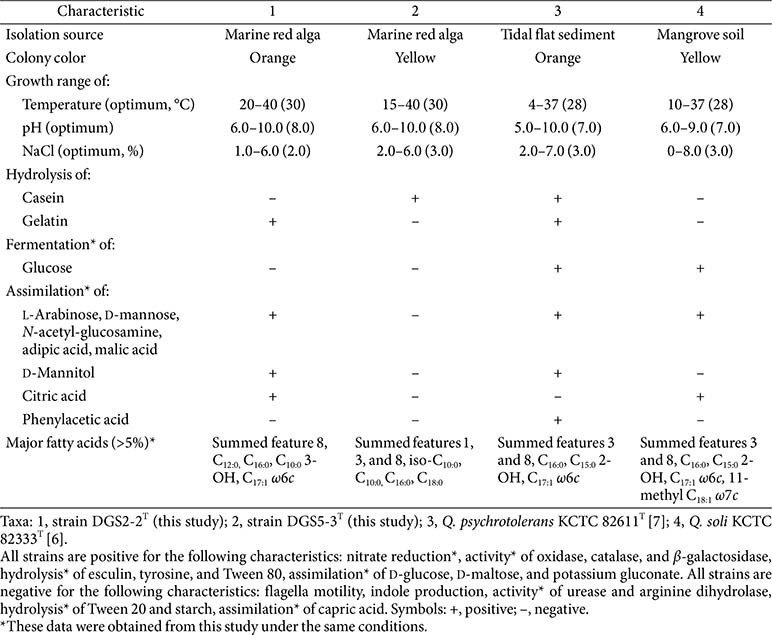# Carotenoid-Producing *Qipengyuania algicola* sp. nov. and *Qipengyuania rhodophyticola* sp. nov., Isolated from Marine Algae, and Emended Description of the Genus *Qipengyuania* Xu *et al*. 2020

**DOI:** 10.4014/jmb.2507.07023

**Published:** 2025-09-16

**Authors:** Jae Kyeong Lee, Min Woo Lee, Chae Yeong Moon, Jeong Min Kim, Hülya Bayburt, Byeong Jun Choi, Che Ok Jeon

**Affiliations:** Department of Life Science, Chung-Ang University, Seoul 06974, Republic of Korea

**Keywords:** *Qipengyuania algicola*, *Qipengyuania rhodophyticola*, new taxa, marine algae, carotenoid pigments

## Abstract

Two Gram-stain-negative, facultatively aerobic, non-motile, catalase- and oxidase-positive, rod-shaped bacteria, designated strains DGS2-2^T^ (orange-pigmented) and DGS5-3^T^ (yellow-pigmented), were isolated from marine red algae collected in Korea. Strain DGS2-2^T^ grew at 20–40°C, pH 6.0–10.0, and in 1.0–6.0% (w/v) NaCl, while DGS5-3^T^ grew at 15–40°C, pH 6.0–10.0, and in 2.0–6.0% NaCl. Ubiquinone-10 was the sole respiratory quinone. The G+C contents were 62.5% for DGS2-2^T^ and 57.5% for DGS5-3^T^. Both strains contained summed feature 8 (C_18:1_*ω*7*c* and/or C_18:1_*ω*6*c*) and C_16:0_ as major fatty acids, and phosphatidylcholine, sphingoglycolipid, phosphatidylethanolamine, phosphatidylglycerol, and diphosphatidylglycerol as major polar lipids. The 16S rRNA gene similarity (97.3%), average nucleotide identity (ANI, 72.0%), and digital DNA-DNA hybridization (dDDH, 18.4%) values between the two strains were below the species delineation thresholds. Phylogenetic and phylogenomic analyses based on 16S rRNA gene and whole-genome sequences placed both strains in distinct lineages within the genus *Qipengyuania*. ANI and dDDH values between each strain and *Qipengyuania* type strains were below 74.5% and 19.5%, respectively, supporting their designation as novel species. Genomic analyses identified putative genes associated with potential algal symbiotic traits, including the biosynthesis of vitamins, siderophores, and hormone-like compounds. Carotenoid biosynthetic genes were also identified, and LC/MS confirmed astaxanthin (DGS2-2^T^) and nostoxanthin (DGS5-3^T^) production. Based on genomic, phylogenetic, phenotypic, and chemotaxonomic evidence, strains DGS2-2^T^ and DGS5-3^T^ represent two novel species of *Qipengyuania*, for which the names *Qipengyuania algicola* sp. nov. (DGS2-2^T^ =KACC 23855^T^ =JCM 37496^T^) and *Qipengyuania rhodophyticola* sp. nov. (DGS5-3^T^ =KACC 23854^T^ =JCM 37497^T^) are proposed.

## Introduction

The genus *Qipengyuania*, belonging to the family *Erythrobacteraceae* within the phylum *Pseudomonadota*, was first proposed in 2015, with *Qipengyuania sediminis* designated as the type species [1]. Phylogenomic analyses of core genes and genome similarity metrics have led to the reclassification of several species from other genera within the family *Erythrobacteraceae* into *Qipengyuania*, resulting in an emended description of the genus [2]. As of July 3, 2025, the genus comprises 35 validly published species and one invalidly published species (https://lpsn.dsmz.de/genus/qipengyuania); however, the LPSN database has not yet incorporated the recent taxonomic revision in which *Qipengyuania aerophila* was reclassified as a later heterotypic synonym of *Qipengyuania pacifica* [3]. Members of *Qipengyuania* have been isolated from a wide range of environments, including subterrestrial sediments [1], deep-sea habitats [4], hot springs [5], mangrove soils [6, 7], tidal flats [8], seawater [9-12], and marine organisms [13]. Cells of *Qipengyuania* are typically Gram-stain-negative, non-spore-forming, aerobic or facultatively aerobic, and chemoorganotrophic, with rod-shaped, spherical, or pleomorphic coccoid morphologies and genomic DNA G+C contents ranging from 60.6% to 66.7% [1, 2, 4-13]. They exhibit catalase-positive and oxidase-variable activities and contain ubiquinone-10 (Q-10) as the predominant respiratory quinone, summed feature 8 (C_18:1_ *ω*7*c* and/or C_18:1_ *ω*6*c*) as the major fatty acids, and phosphatidylcholine (PC), phosphatidylethanolamine (PE), phosphatidylglycerol (PG), diphosphatidylglycerol (DPG) as the major polar lipids. *Qipengyuania* species characteristically produce yellow to orange carotenoid pigments through gene clusters encoding the biosynthesis of compounds such as astaxanthin, canthaxanthin, nostoxanthin, *β*-carotene, and zeaxanthin, which may provide photoprotection and oxidative stress resistance to the bacteria and their hosts, and have potential biotechnological applications in the food, pharmaceutical, and cosmetic industries [3, 4, 6, 7]. During our investigation of bacterial communities associated with marine algae [14-17], we isolated two putative novel *Qipengyuania* strains from the algal phycosphere. In this study, we present their taxonomic characterization using a polyphasic approach and evaluate their carotenoid pigment-producing potential.

## Material and Methods

### Isolation and Cultivation

Strains DGS2-2^T^ and DGS5-3^T^ were isolated from the marine red algae *Gracilariopsis* sp. and *Chondrus* sp., respectively, which were collected from Donggo-ri Beach, South Korea (34°19'37.0''N, 126°52'47.0''E), as previously reported with slight modifications [17]. In brief, the algal samples were rinsed in sterilized artificial seawater (ASW) and then homogenized for 1 minute using a T10 basic homogenizer (IKA, Germany). The samples were serially diluted in ASW, and 100 μl of each dilution was spread onto marine agar (MA; MBcell, South Korea), followed by aerobic incubation at 25°C for 3 days. Colonies were identified through PCR amplification of the 16S rRNA gene with primers 27F and 1492R [17]. The amplicons were digested with restriction enzymes HaeIII and HhaI, then separated by 2% (w/v) agarose gel electrophoresis. The distinct patterns of representative products were sequenced partially using primer 340F [17]. The resulting sequences were compared to those of type strains via EzBioCloud (http://www.ezbiocloud.net/) [18]. Two strains, identified as potential members of the genus *Qipengyuania*, were selected for further phenotypic and phylogenetic analysis. These strains were routinely cultured on MA at 30°C for 3 days and stored at –80°C in marine broth (MB; MBcell) containing 15% (v/v) glycerol. Based on their phylogenetic positions in the 16S rRNA gene tree, *Qipengyuania psychrotolerans* KCTC 82611^T^ and *Qipengyuania soli* KCTC 82333^T^ were chosen as reference strains for comparative analyses of genomic features, phenotypic traits, and fatty acid compositions.

### Phylogenetic Analysis Based on 16S rRNA Gene Sequences

The 16S rRNA gene amplicons from strains DGS2-2^T^ and DGS5-3^T^, initially amplified using primers 27F and 1492R, were subsequently sequenced with the universal primers 518R and 805F [17]. Sequences generated with primers 340F, 518R, and 805F were assembled, and their similarity to sequences of closely related type strains was assessed using the EzBioCloud platform. Multiple sequence alignments of the 16S rRNA gene sequences were conducted with Infernal v1.1.4, utilizing the Rfam covariance model RF00177 [19]. Phylogenetic trees were constructed using the neighbor-joining (NJ), maximum-parsimony (MP), and maximum-likelihood (ML) approaches in MEGA11 software [20], with 1,000 bootstrap replications. For the tree constructions, the Kimura two-parameter model was applied for NJ, the nearest-neighbor-interchange heuristic search for MP, and pairwise deletion for ML.

### Genome Sequencing and Phylogenomic Analysis

Genomic DNA from strains DGS2-2^T^ and DGS5-3^T^ was extracted from cultures grown in MB using the Wizard Genomic DNA Purification Kit (Promega, USA), following the manufacturer's instructions. Whole-genome sequencing was performed in-house using the Oxford Nanopore MinION platform (ONT, UK), adhering to the manufacturer's protocols. In brief, genomic DNA libraries were prepared using the Ligation Sequencing Kit (SQK-NBD114.24; ONT), loaded onto an R10.4.1 flow cell (ONT), and sequenced for approximately 24 h. Basecalling was carried out with Guppy v6.5.7 in super accurate (SUP) mode. The sequencing reads were assembled de novo using Flye v2.9.1 [21], and genome quality was evaluated using CheckM2 v1.0.2 [22] to assess completeness and contamination. Phylogenomic analysis was performed using GTDB-Tk, based on the concatenated sequences of 120 core single-copy marker genes (bac120 set) [23]. The aligned sequences were employed to construct an ML tree with 1,000 bootstrap replications in MEGA11. Average nucleotide identity (ANI) and digital DNA-DNA hybridization (dDDH) values were calculated using the Orthologous ANI Tool (OAT v0.93.1; www.ezbiocloud.net/tools/orthoani) [24] and the Genome-to-Genome Distance Calculator (GGDC 3.0; https://ggdc.dsmz.de/ggdc.php), respectively, applying formula 2 [25].

### Genomic Features and Functional Gene Annotations

The whole-genome sequences of strains DGS2-2^T^ and DGS5-3^T^ have been deposited in GenBank, with general genomic features and gene annotations, along with those of two closely related reference *Qipengyuania* type strains, retrieved from GenBank annotations generated through the NCBI Prokaryotic Genome Annotation Pipeline. To evaluate the potential for algal polysaccharide degradation in strains DGS2-2^T^ and DGS5-3^T^, as well as the two reference *Qipengyuania* strains, all predicted protein sequences were examined for carbohydrate-active enzymes (CAZys) using the dbCAN3 meta server (https://bcb.unl.edu/dbCAN2/blast.php) [26]. Furthermore, genes potentially associated with symbiotic interactions with marine algae were identified through BLASTP analysis, using reference protein sequences from the UniProt database (https://www.uniprot.org). Since members of the genus *Qipengyuania* are known to produce carotenoid pigments [7, 27], carotenoid biosynthesis gene clusters were identified in strains DGS2-2^T^ and DGS5-3^T^, as well as in the two reference *Qipengyuania* strains, using BLASTP analysis, and their carotenoid biosynthetic pathways were reconstructed.

### Analysis of Carotenoid Pigments

Carotenoid pigments from strains DGS2-2^T^ and DGS5-3^T^ and two reference *Qipengyuania* strains were analyzed following a previously established protocol with minor adjustments [28]. The strains were cultured in MB for 3 days at 30°C in the dark. A 5 ml aliquot of each culture was centrifuged at 12,000 ×*g* for 5 min, and the resulting cell pellets were extracted with 5 ml acetone/methanol (1:1, v/v). The mixtures were incubated overnight at 4°C with shaking at 200 rpm in the dark, then centrifuged at 12,000 ×*g* for 15 min. The supernatants were evaporated under nitrogen, and the dried pigments were reconstituted in 200 μl methanol, filtered through a 0.45 μm PVDF syringe filter (Millipore, USA), and analyzed for absorption spectra using a Synergy H1 plate reader (BioTek, USA).

Carotenoid pigments from *Qipengyuania* strains were analyzed using an LC-QTOF-MS system, which included a diode array detector (DAD), a 1290 Infinity UHPLC, and a 6550 iFunnel Q-TOF mass spectrometer (Agilent Technologies, USA), following a modified protocol [29]. In brief, 5 μl of the extract was injected into an Agilent Eclipse Plus C18 column (2.1 mm × 100 mm, 2.1 μm) maintained at 40°C. The mobile phases were water (A) and acetonitrile (B), both containing 0.1% formic acid, at a flow rate of 0.6 ml/min, with the following gradient: 0–1.0 min, 60% A/40% B; 1.0–16.0 min, a linear transition to 0% A/100% B; 16.0–18.0 min, held at 100% B; 19.0 min, return to initial conditions; 20 min for re-equilibration. Mass spectrometry was conducted in positive ion mode with a gas temperature of 275°C, a nebulizer pressure of 30 psi, a capillary voltage of +4,000 V, an MS range of 50–1,500 m/z, and an MS/MS range of 30–1,500 m/z, with a collision energy of 20 eV. Chromatograms were recorded at 475 nm, and UV-V is spectra (300–600 nm) of prominent peaks were collected. Accurate mass measurements were obtained using internal reference ions (m/z 121.050873 and 922.009798). Carotenoids were identified using the “find by formula” function in Agilent MassHunter Qualitative Analysis B10.0.

### Phenotypic, Physiological, and Biochemical analyses

The growth of strains DGS2-2^T^ and DGS5-3^T^ was assessed on various agar media (all from MBcell), including MA, R2A agar, tryptic soy agar (TSA), nutrient agar (NA), and Luria-Bertani (LB) agar, each supplemented with approximately 2% (w/v) NaCl, and incubated at 30°C for 3 days. The temperature range for growth was tested on MA at temperatures ranging from 10–45°C in 5°C increments. pH tolerance was evaluated in MB adjusted to pH values of 4.0–11.0 (in 1.0-unit increments) at 25°C for 3 days. pH-adjusted media were prepared using sodium citrate (for pH 4.0–5.0), sodium phosphate (for pH 6.0–8.0), or sodium carbonate–bicarbonate buffers (for pH 9.0–11.0), with pH adjustments made post-autoclaving as needed. Salt tolerance was determined in MB containing 0–10% (w/v) NaCl (in 1.0% increments) at 30°C for 3 days. Anaerobic growth was tested on MA after 21 days at 30°C under anaerobic conditions created using the GasPak Plus system (BBL, USA).

Physiological and biochemical tests were carried out using cells cultured on MA at 30°C for 3 days. Cell morphology and motility were observed under a phase-contrast microscope (Axio Scope.A1; Carl Zeiss, Germany). For ultrastructural and flagellar analysis, cells were negatively stained with 2% (w/v) uranyl acetate on formvar-coated copper grids and examined with a transmission electron microscope (JEM-1010; Jeol, Japan). Motility was further evaluated by stab-inoculating MA containing 0.3% (w/v) agar and incubating at 30°C. Gram staining was performed using a commercial kit (bioMérieux, France). Catalase activity was assessed by the formation of bubbles in 3% (v/v) hydrogen peroxide (Junsei, Japan), while oxidase activity was evaluated by color change with 1% (w/v) tetramethyl-*p*-phenylenediamine (Merck, USA) [30]. The phenotypic characteristics of strains DGS2-2^T^ and DGS5-3^T^ were compared with those of two closely related reference strains under the same conditions. Hydrolysis of tyrosine, casein, urea, esculin, starch, gelatin, Tween 20, and Tween 80 was tested on MA as previously described [30]. Additional biochemical traits were assessed using the API 20NE system (bioMérieux, France), with ASW as the suspension medium.

### Chemotaxonomic Analyses

Respiratory isoprenoid quinones from strains DGS2-2^T^ and DGS5-3^T^ were extracted from cells cultured in MB for 3 days at 30°C and analyzed using an HPLC system (LC-20A, Shimadzu, Japan) fitted with a reversed-phase column (250 × 4.6 mm, Kromasil, Akzo Nobel, Netherlands) and a diode array detector (SPD-M20A, Shimadzu). The mobile phase consisted of a methanol-isopropanol mixture (2:1, v/v) at a flow rate of 1 ml/min, following a previously established method [31]. For fatty acid analysis, the two strains and two reference strains were grown aerobically in MB at their optimal growth temperatures and collected during the exponential phase (OD_600_ =0.7–0.8). Fatty acid methyl esters were prepared according to the standard protocol of the Sherlock Microbial Identification System (MIDI, version 6.2B), which includes saponification, methylation, and extraction, and analyzed with a Hewlett Packard 6890 gas chromatograph. Fatty acids were identified using the RTSBA6 database (Sherlock version 6.0B) [32].

Polar lipids from strains DGS2-2^T^ and DGS5-3^T^, as well as two closely related reference strains, were analyzed using two-dimensional thin-layer chromatography. Cells were collected during the exponential growth phase, and polar lipids were extracted and separated according to a previously outlined method [33]. Various staining reagents were employed to identify different lipid classes: 10% ethanolic molybdophosphoric acid for total lipids, ninhydrin for aminolipids, Dittmer-Lester reagent for phospholipids, and α-naphthol/sulfuric acid for glycolipids. The presence of PC, PE, PG, and DPG was verified using standard polar lipid compounds (Sigma-Aldrich, USA).

## Results and Discussion

### Strain Isolation and Phylogeny Based on 16S rRNA Gene Sequences

To prevent the repeated isolation of identical bacterial strains from marine red algae, 16S rRNA genes of colonies grown on MA were PCR-amplified, digested with HaeIII and HhaI, and categorized based on their fragment patterns. Colonies exhibiting distinct fragment patterns were sequenced and subjected to phylogenetic analysis using the EzBioCloud platform. Strains DGS2-2^T^ and DGS5-3^T^, identified as members of the genus *Qipengyuania*, were successfully isolated from the phycosphere of marine red algae. Their nearly complete 16S rRNA gene sequences (1,411 bp each) were assembled from reads generated with primers 340F, 518R, and 805F.

The 16S rRNA gene sequence similarity between the two strains was 97.3%, which is below the typical species-level threshold of 98.5–98.7% [34], indicating that they are distinct species. Strain DGS2-2^T^ exhibited the highest similarity to *Qipengyuania citrea* RE35F/1^T^ (97.6%) and *Q. psychrotolerans* 1XM2-8^T^ (97.5%), whereas strain DGS5-3^T^ showed the greatest similarity to *Q. psychrotolerans* 1XM2-8^T^ and *Q. citrea* RE35F/1^T^ (both 98.4%). Since all similarity values are below the species delineation threshold, these strains are likely novel species within the genus *Qipengyuania*, based on comparisons of their 16S rRNA gene sequences.

**Fig. 1:**
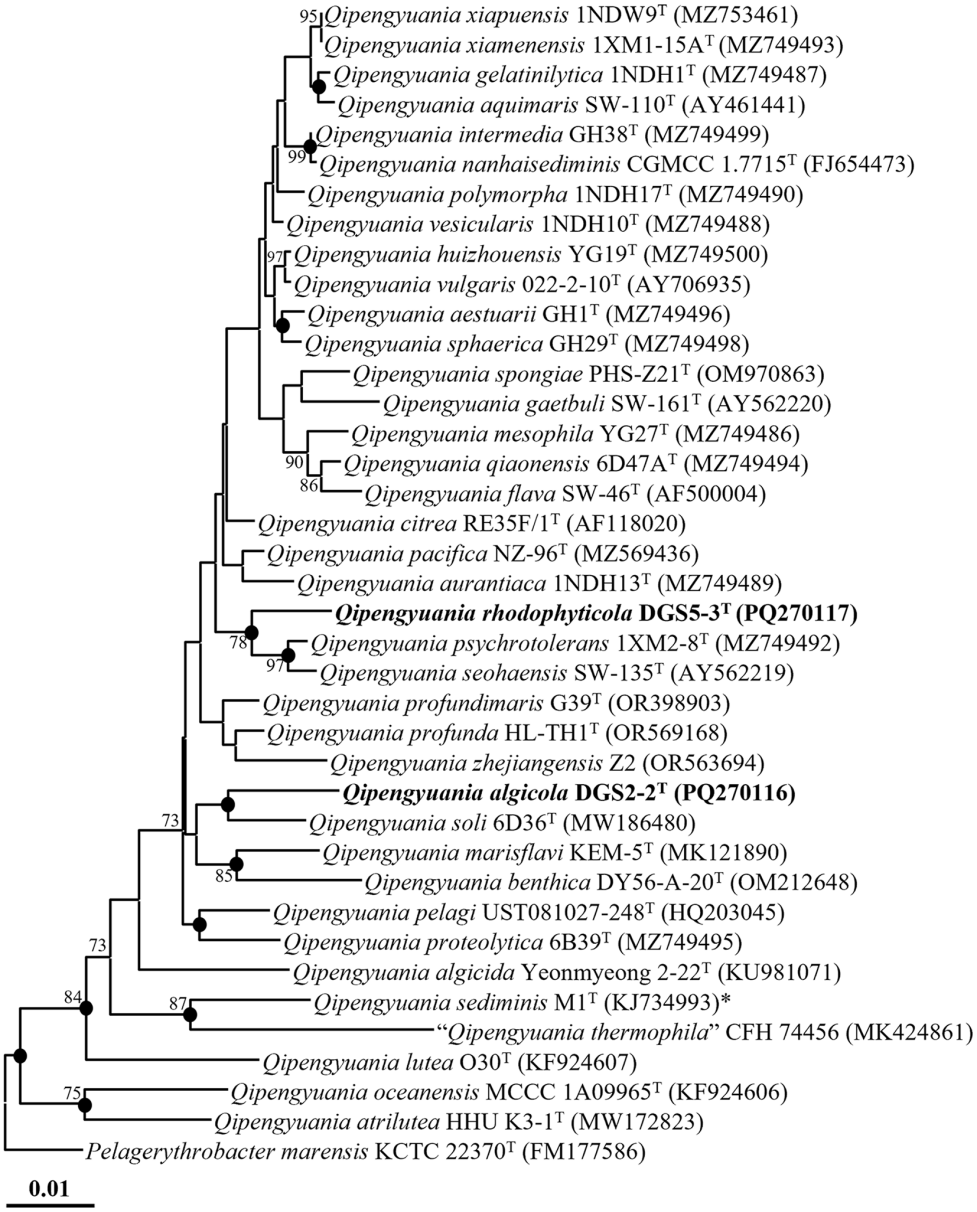
Neighbor-joining tree showing the phylogenetic relationships between strains DGS2-2^T^ and DGS5-3^T^ and closely related species, based on 16S rRNA gene sequences. Bootstrap values (>70%) from 1,000 replicates are shown at branch nodes. Filled circles (l) indicate nodes also supported by maximum-likelihood and maximum-parsimony trees, and the type species of the genus *Qipengyuania* is denoted with an asterisk (*). *Pelagerythrobacter marensis* KCTC 22370^T^ (FM177586) was used as the outgroup. Scale bar, 0.01 substitutions per nucleotide.

Phylogenetic analysis based on 16S rRNA gene sequences, using the NJ method, revealed that strain DGS2-2^T^ clustered with *Q. soli* 6D36^T^, while strain DGS5-3^T^ formed a group with *Q. psychrotolerans* 1XM2-8^T^ and *Qipengyuania seohaensis* SW-135^T^, within the *Qipengyuania* clade (Fig. 1). These relationships were consistently supported by phylogenetic trees generated with the ML and MP methods (Fig. S1), confirming that both strains belong to the genus *Qipengyuania*. Taken together, the sequence similarity and phylogenetic evidence strongly suggest that strains DGS2-2^T^ and DGS5-3^T^ represent two novel species within the genus *Qipengyuania*.

### Whole Genome Sequencing, Phylogeny Based on Genome Sequences, and Genome Relatedness

*De novo* assembly of MinION sequencing reads, with average genome coverages of 85× for strain DGS2-2^T^ and 111× for strain DGS5-3^T^, resulted in complete genomes of approximately 2,886 kb and 3,093 kb, respectively. Genome quality evaluation revealed 100% completeness and 0.1% contamination for both strains, meeting the established standards for high-quality genomes (≥90% completeness and ≤10% contamination) [22]. The genomic G+C contents were 62.5% for DGS2-2^T^ and 57.5% for DGS5-3^T^. The G+C content of DGS2-2^T^ is within the previously reported range for the genus *Qipengyuania* (60.6–66.7%) [2], while DGS5-3^T^ exhibits a notably lower value. Other species within the genus *Qipengyuania*, such as *Q. sediminis* (73.7%) and *Q. psychrotolerans* (60.1%) [1, 15], also show variations that deviate from the typical genus range. These observations suggest that the genus *Qipengyuania* may need to be redefined to encompass the broader G+C content variation seen among its members.

**Fig. 2:**
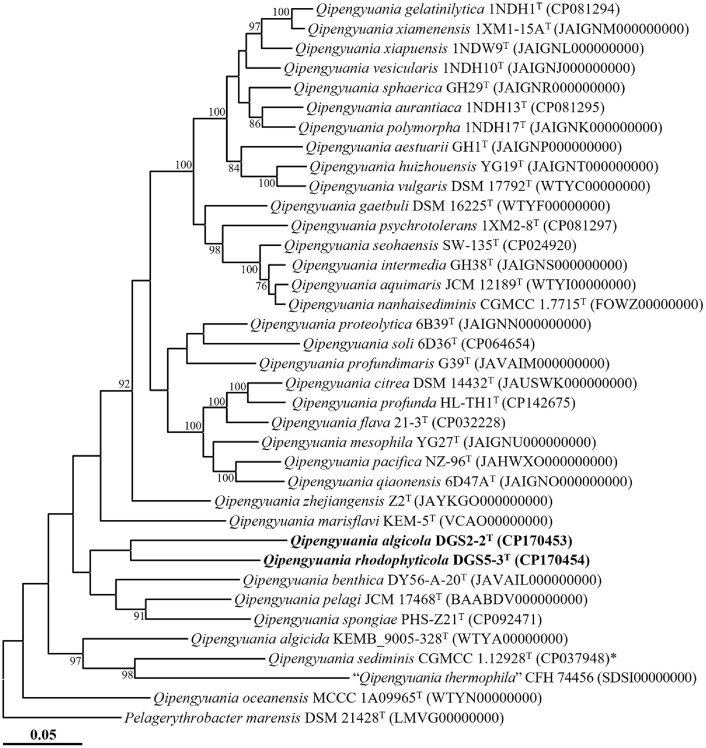
Maximum-likelihood phylogenomic tree showing the phylogenetic relationships between strains DGS2-2^T^ and DGS5-3^T^ and closely related taxa, based on the concatenated amino acid sequences of 120 bacterial marker genes (bac120 marker set) of GTDB-Tk. Bootstrap values (>70%) from 1,000 replicates are shown at branch nodes. *Pelagerythrobacter marensis* DSM 21428^T^ (LMVG00000000) was employed as an outgroup, and the type species of the genus *Qipengyuania* is denoted with an asterisk (*). Scale bar, 0.05 changes per amino acid position.

The phylogenomic tree based on genome data showed that strains DGS2-2^T^ and DGS5-3^T^ formed separate phylogenetic lineages within the genus *Qipengyuania*, as depicted in Fig. 2. This finding aligns with their classification within the genus based on 16S rRNA gene sequence analysis. The ANI and dDDH values between the two strains were 72.0% and 18.4%, respectively, both of which are significantly below the typical species delineation thresholds (ANI ~95%; dDDH ~70%) [34], suggesting that they represent distinct species. Furthermore, the ANI and dDDH values between each strain and all other known *Qipengyuania* species were below 72.7% and 19.5%, respectively (Table S1), providing additional evidence that they are separate from previously described taxa. Collectively, these genome-based phylogenomic and similarity analyses provide strong support for the classification of strains DGS2-2^T^ and DGS5-3^T^ as two new species within the genus *Qipengyuania*.

### Genomic Features and Functional Gene Analysis

The genome of strain DGS2-2^T^ consists of a single circular chromosome of 2,886,478 bp, which encodes 2,748 genes, including 2,693 protein-coding sequences, one rRNA operon (16S, 23S, 5S), 43 tRNA genes, and four noncoding RNA genes. In a similar manner, the genome of strain DGS5-3^T^ contains a single circular chromosome of 3,092,645 bp with 2,936 genes, comprising 2,878 protein-coding sequences, one rRNA operon, 41 tRNA genes, and four noncoding RNA genes. The general genomic characteristics of strains DGS2-2^T^ and DGS5-3^T^ closely resemble those of related *Qipengyuania* type strains and are summarized in Table 1.

Marine algae are primarily composed of polysaccharides, and the ability to break down these compounds may be a crucial characteristic for heterotrophic bacteria to thrive in the algal phycosphere. To explore this, the CAZy genes of strains DGS2-2^T^ and DGS5-3^T^, isolated from the phycosphere of marine algae, were examined. The genomes of DGS2-2^T^ and DGS5-3^T^ were found to encode 56 and 54 CAZy-related genes, respectively, which is similar to the CAZy gene content of closely related *Qipengyuania* strains *Q. psychrotolerans* 1XM2-8^T^ and *Q. soli* 6D36^T^, although these strains were not isolated from the algal phycosphere (Table 1). However, the number of CAZy genes in these strains is relatively lower compared to other bacteria associated with marine algae [15, 35, 36]. Notably, strains DGS2-2^T^ and DGS5-3^T^ each possess two and five polysaccharide lyase genes, respectively—enzymes from the CAZy family directly involved in polysaccharide degradation—which are absent in *Q. psychrotolerans* and *Q. soli*. This suggests that the capacity to degrade algal polysaccharides through polysaccharide lyases may be an essential adaptive feature for the survival and success of strains DGS2-2^T^ and DGS5-3^T^ in the algal phycosphere.

Bacteria inhabiting the algal phycosphere can aid their hosts through various metabolic interactions, including the production of vitamins, hormone-like substances, siderophores, and other essential nutrients [29]. Genomic analysis of strains DGS2-2^T^ and DGS5-3^T^ identified the presence of biosynthetic gene clusters for compounds that may benefit marine algae. Both strains contain the putative *thiCDE-phoA* and *ribABDEH* gene clusters, which are involved in synthesizing thiamine (vitamin B_1_) from aminoimidazole ribotide and riboflavin (vitamin B_2_) from guanosine 5'-triphosphate (GTP) and ribulose-5-phosphate, respectively (Fig. S2). However, neither strain carries the phosphatase genes (*ybiI*, *ycsE*, *yigB*) required to convert 5-amino-6-(5'-phospho-d-ribitylamino)uracil to 5-amino-6-(d-ribitylamino)uracil (Fig. S2), suggesting they may not produce riboflavin independently but rather synthesize it in cooperation with other organisms. Furthermore, both strains possess a complete set of genes (*folBCEKP* and *phoD*) potentially responsible for the synthesis of dihydrofolate, a precursor of folate (vitamin B_9_), from GTP (Fig. S3). Both strains also contain key genes involved in producing phenylacetic acid (*katG* and *amiE*) and 2-hydroxy-phenylacetic acid (*hisC* and *hppD*), hormone-like compounds derived from L-phenylalanine (Fig. S4), which are thought to enhance algal growth and stress tolerance [29]. Additionally, both strains harbor the putative *bfr* gene (locus tag: ACFCW2_02540 for DGS2-2^T^ and ACFCXQ_00805 for DGS5-3^T^), which encodes bacterioferritin, a siderophore-related protein involved in iron acquisition, a key function for promoting algal growth in marine environments [37]. These metabolic traits in strains DGS2-2^T^ and DGS5-3^T^ suggest they may play a role in supporting symbiotic relationships with marine algae and potentially enhancing algal growth.

**Fig. 3:**
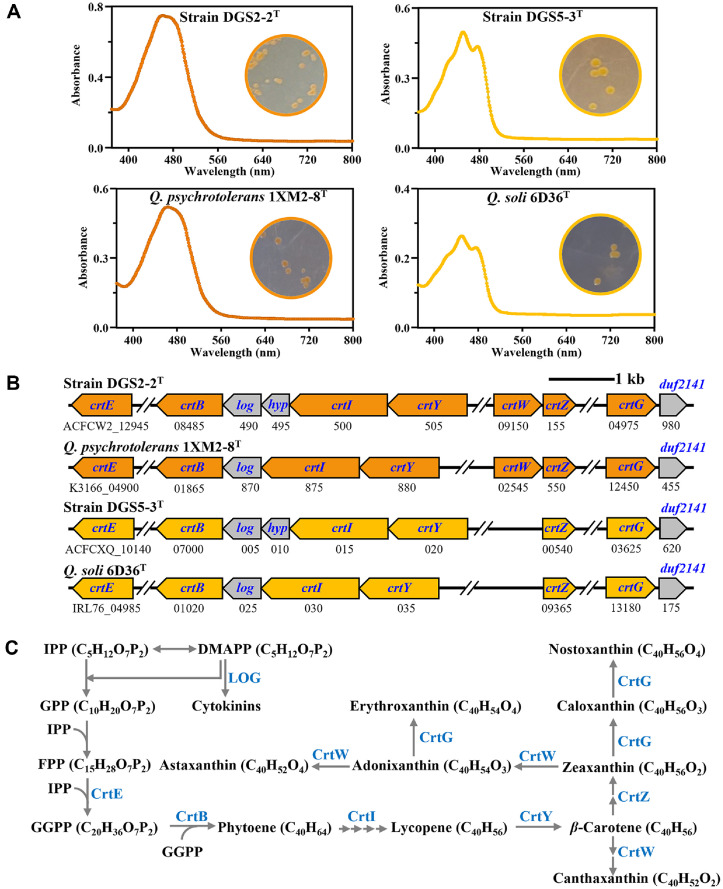
Carotenoid pigment production (A) carotenoid biosynthesis-related genes (B) and proposed biosynthetic pathways (C) identified in strains DGS2-2^T^ and DGS5-3^T^, along with their closely related *Qipengyuania* strains, *Q. psychrotolerans* 1XM2-8^T^ and *Q. soli* 6D36^T^. The UV-visible spectra in panel A were obtained from methanol extracts of cultured cells, with colony photographs showing pigmentation presented as insets. Gene annotations are as follows: *crtE*, polyprenyl synthetase; *crtB*, phytoene synthase; *crtI*, phytoene desaturase; *crtY*, lycopene *β*-cyclase; *crtG*, 2,2'-*β*-hydroxylase; *crtW*, *β*-carotene ketolase; *crtZ*, *β*-carotene hydroxylase; *log*, lonely guy (cytokinin phosphoribohydrolase); *hyp*, hypothetical protein; *duf*, domain of unknown function. Abbreviations: IPP, isopentenyl pyrophosphate; DMAPP, dimethylallyl pyrophosphate; GPP, geranyl pyrophosphate; FPP, farnesyl pyrophosphate; GGPP, geranylgeranyl pyrophosphate.

**Fig. 4:**
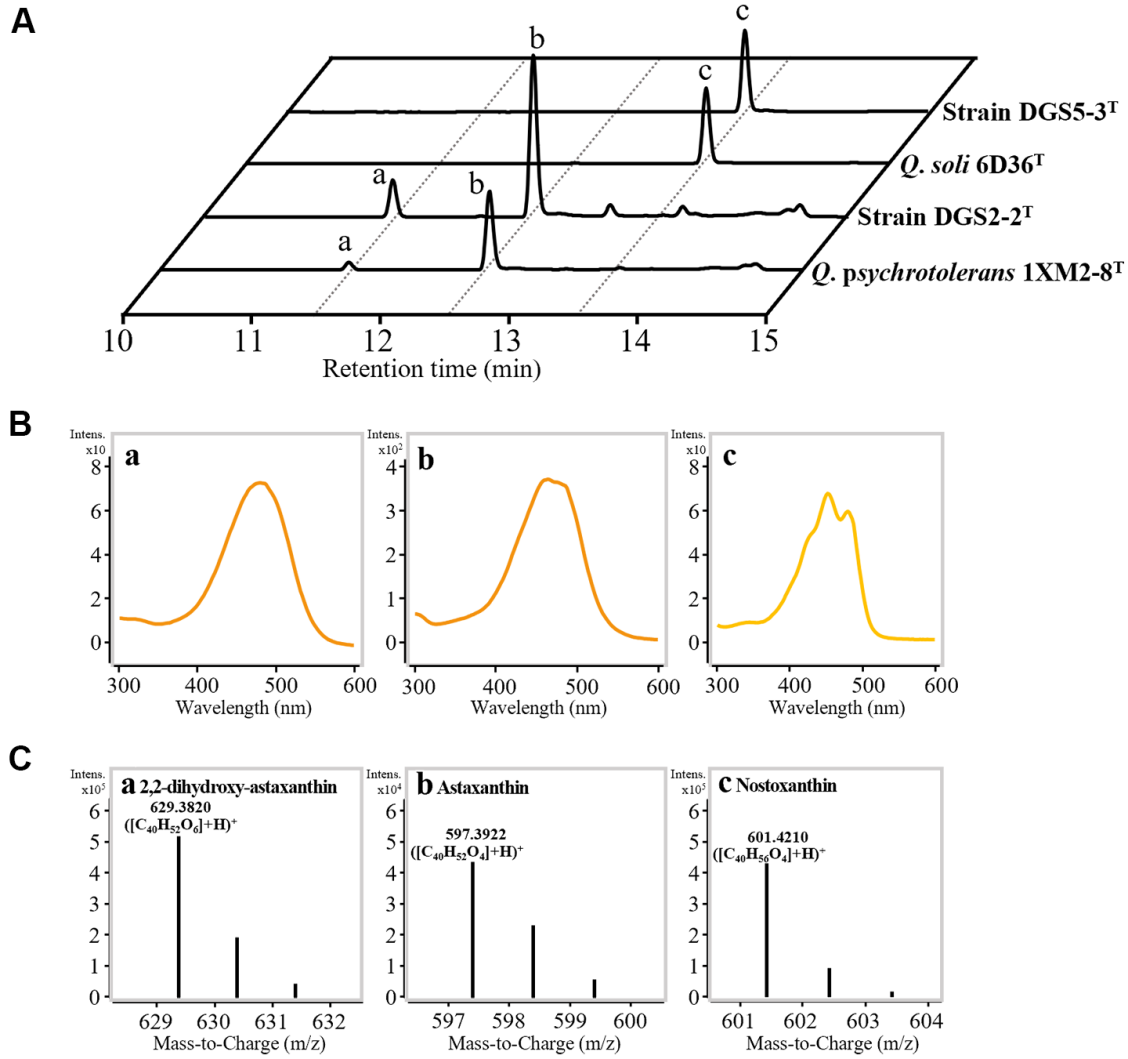
Liquid chromatograms of methanol extracts from cultured cells of strains DGS2-2^T^ and DGS5-3^T^, along with their closely related *Qipengyuania* strains (*Q. psychrotolerans* 1XM2-8^T^ and *Q. soli* 6D36^T^), monitored at 475 nm (A) and UV-Vis (B) and mass spectra (C) of major peaks obtained using LC-Q-TOFMS with a diode array detector. Carotenoid compounds were predicted based on chemical formulas derived from LC-QTOF- MS and the proposed biosynthetic pathways shown in Fig. 3C.

Strains DGS2-2^T^ and DGS5-3^T^ formed colonies with orange and yellow pigmentation, respectively, and their methanol extracts displayed characteristic carotenoid absorption spectra between 380 and 530 nm, confirming the production of carotenoids (Fig. 3A). Their genomes were then examined for carotenoid biosynthesis genes (Fig. 3B), revealing gene clusters similar to those found in other *Qipengyuania* species [7]. Strain DGS2-2^T^, which exhibited orange pigmentation, contains the *crtE*, *crtB*, *crtI*, *crtY*, *crtW*, *crtZ*, and *crtG* genes, a profile largely consistent with that of *Q. psychrotolerans* 1XM2-8^T^, with the exception of a hypothetical protein gene (*hyp*) located downstream of *crtI*. Based on these gene profiles, both strain DGS2-2^T^ and *Q. psychrotolerans* 1XM2-8^T^ are likely capable of producing a variety of carotenoids, including astaxanthin, erythroxanthin, canthaxanthin, nostoxanthin, *β*-carotene, and zeaxanthin (Fig. 3C). However, their orange pigmentation and strong absorbance in the 450–500 nm range suggest that the predominant carotenoids are likely to be orange carotenoids such as astaxanthin, erythroxanthin, and canthaxanthin. In contrast, strain DGS5-3^T^, which showed yellow pigmentation, possesses *crtE*, *crtB*, *crtI*, *crtY*, *crtZ*, and *crtG*, a gene profile similar to that of *Q. soli* 6D36^T^, with the addition of a *hyp* gene downstream of *crtI*. Notably, both strain DGS5-3^T^ and *Q. soli* 6D36^T^ lack *crtW* (*β*-carotene ketolase), which prevents the synthesis of orange carotenoids such as astaxanthin, erythroxanthin, canthaxanthin, or adonixanthin, explaining their yellow pigmentation. To identify the carotenoid compounds produced by these strains, methanol extracts from cultured cells of DGS2-2^T^, DGS5-3^T^, *Q. psychrotolerans* 1XM2-8^T^, and *Q. soli* 6D36^T^ were analyzed using LC-Q-TOF-MS. Astaxanthin was predominantly detected in strain DGS2-2^T^ and *Q. psychrotolerans* 1XM2-8^T^, with 2,2'-dihydroxy-astaxanthin, likely formed from astaxanthin via *crtG* (2,2'-*β*-hydroxylase), identified as a minor component, consistent with their orange pigmentation (Fig. 3A). In contrast, only nostoxanthin was found in strain DGS5-3^T^ and *Q. soli* 6D36^T^ (Fig. 4), corresponding to the absence of the *crtW* gene (Fig. 3B) and their yellow pigmentation (Fig. 3A).

### Phenotypic, Physiological, and Biochemical Characteristics

Both strains DGS2-2^T^ and DGS5-3^T^ demonstrated strong growth on MA. Strain DGS2-2^T^ grew slowly on TSA supplemented with approximately 2% NaCl but failed to grow on R2A agar, LB agar, or NA with similar NaCl concentrations. In contrast, strain DGS5-3^T^ did not grow on any of these media. The cells of both strains were Gram-negative rods without flagella, measuring 0.5–0.6 μm in width and 1.1–1.2 μm in length for strain DGS2-2^T^, and 0.5–0.6 μm in width and 0.9–1.0 μm in length for strain DGS5-3^T^ (Fig. S5). Both strains showed slight growth on MA under anaerobic conditions after 21 days of incubation, indicating their facultative aerobic metabolism. A range of phenotypic characteristics, such as rod-shaped morphology, absence of flagellar motility, nitrate reduction (+), indole production (–), oxidase activity (+), catalase activity (+), *β*-galactosidase activity (+), urease (–), arginine dihydrolase (–), and hydrolysis of esculin (+), tyrosine (+), and Tween 80 (+), but not starch (–) or Tween 20 (–), were consistent with those observed for reference strains of the genus *Qipengyuania* (Table 2). However, differences in other phenotypic traits—such as the growth temperature range, hydrolysis of casein and gelatin, glucose fermentation, and the assimilation of L-arabinose, D-mannose, *N*-acetyl-glucosamine, adipic acid, malic acid, D-mannitol, citric acid, and phenylacetic acid—distinguished strains DGS2-2^T^ and DGS5-3^T^ from closely related *Qipengyuania* species.

### Chemotaxonomic Characteristics

The only respiratory isoprenoid quinone detected in both strains DGS2-2^T^ and DGS5-3^T^ was Q-10, which aligns with the predominant quinone found in other *Qipengyuania* species [1, 2, 4-13]. The major cellular fatty acids (comprising more than 5% of the total) in both strains included summed feature 8 (comprising C_18:1_ *ω*7*c* and/or C_18:1_ *ω*6*c*) and C_16:0_, which is consistent with those found in related *Qipengyuania* strains (Table S2). Additionally, strain DGS2-2^T^ contained C_12:0_, C_17:1_ *ω*6*c*, and C_10:0_ 3-OH, whereas strain DGS5-3^T^ exhibited C_10:0_, C_18:0_, iso-C_10:0_, and summed features 1 (iso-C_15:1_ H and/or C_13:0_ 3-OH) and 3 (C_16:1_ *ω*7*c* and/or C_16:1_ *ω*6*c*) as major fatty acids. While both strains shared summed feature 8 and C_16:0_ as dominant fatty acids, their overall fatty acid profiles differed from each other and from those of other *Qipengyuania* type strains. The exclusive presence of C_10:0_ 3-OH in DGS2-2^T^ and iso-C_10:0_ in DGS5-3^T^ further distinguishes them from each other and from related taxa. The major polar lipids identified in both strains included PC, sphingoglycolipid (SGL), PE, PG, and DPG, along with some unidentified polar lipids (Fig. S6), which are generally in agreement with the lipid profiles of other *Qipengyuania* type strains [1, 4-13].

### Taxonomic Conclusion

In summary, phylogenetic analysis, genomic relatedness, and the assessment of phenotypic, biochemical, and chemotaxonomic traits consistently indicate that strains DGS2-2^T^ and DGS5-3^T^ represent two distinct, novel species within the genus Qipengyuania. Therefore, we propose the names *Qipengyuania algicola* sp. nov. for strain DGS2-2^T^ and *Qipengyuania rhodophyticola* sp. nov. for strain DGS5-3^T^.

### Description of *Qipengyuania algicola* sp. nov.

*Qipengyuania algicola* (al.gi'co.la. L. fem. n. *alga*, an alga; L. suffix. -*cola*, (from L. masc. or fem. n. *incola*), inhabitant, dweller; N.L. fem. n. algicola, an alga dweller).

Cells are Gram-stain-negative, facultatively aerobic, and non-motile short rods. Colonies on MA agar are orange, circular, and convex. Gliding motility is not observed. Growth occurs at 20–40°C (optimum, 30°C), pH 6.0–10.0 (optimum, 8.0), and 1.0–6.0% (w/v) NaCl (optimum, 2.0% NaCl). Catalase- and oxidase-positive. Esculin, tyrosine, Tween 80, and gelatin are hydrolyzed, but Tween 20, starch, and casein are not. Nitrate reduction is positive, but fermentation of D-glucose and indole production are negative. Positive for *β*-galactosidase activity, but negative for urease and arginine dihydrolase activities. Assimilation of D-glucose, D-maltose, L-arabinose, D-mannose, D-mannitol, *N*-acetylglucosamine, potassium gluconate, adipic acid, malic acid, and citric acid is positive, but assimilation of capric acid and phenylacetic acid is negative. The major cellular fatty acids (>5%) are summed feature 8 (comprising C_18:1_ *ω*7*c* and/or C_18:1_ *ω*6*c*), C_17:1_ *ω*6*c*, C_16:0_, C_10:0_ 3-OH, and C_12:0_. PC, SGL, PE, PG, DPG are identified as major polar lipids. The DNA G+C content calculated from the whole genome sequence of the type strain is 62.5 mol%.

The type strain is DGS2-2^T^ (=KACC 23855^T^ =JCM 37496^T^), isolated from the marine red alga *Gracilariopsis* sp. collected at Donggo-ri Beach, Republic of Korea. The GenBank accession numbers for the 16S rRNA gene and whole-genome sequences of strain DGS5-3^T^ are PQ270116 and CP170453, respectively.

### Description of *Qipengyuania rhodophyticola* sp. nov.

*Qipengyuania rhodophyticola* (rho.do.phy.ti'co.la. N.L. neut. pl. n. *Rhodophyta*, the division of the red algae; L. suffix. -*cola* (from L. masc. or fem. n. *incola*), inhabitant, dweller; N.L. fem. n. *rhodophyticola*, inhabitant of *Rhodophyta*).

Cells are Gram-stain-negative, facultatively aerobic, and non-motile short rods. Colonies on MA agar are yellow, circular, and convex. Gliding motility is not observed. Growth occurs at 15–40°C (optimum, 30°C), pH 6.0–10.0 (optimum, 8.0), and 2.0–6.0% (w/v) NaCl (optimum, 3.0% NaCl). Catalase- and oxidase-positive. Esculin, tyrosine, Tween 80, casein are hydrolyzed, but Tween 20, starch, and gelatin are not. Nitrate reduction is positive, but fermentation of D-glucose and indole production is negative. Positive for *β*-galactosidase activity, but negative for urease and arginine dihydrolase activities. Assimilation of D-glucose, D-maltose, and potassium gluconate is positive, but assimilation of L-arabinose, D-mannose, D-mannitol, *N*-acetylglucosamine, adipic acid, malic acid, citric acid, capric acid, and phenylacetic acid is negative. The major cellular fatty acids (>5%) are summed feature 8 (comprising C_18:1_ *ω*7*c* and/or C_18:1_ *ω*6*c*), summed feature 3 (comprising C_16:1_ *ω*7*c* and/or C_16:1_ *ω*6*c*), C_16:0_, C_10:0_, C_18:0_, summed feature 1 (comprising iso-C_15:1_ H and/or C_13:0_ 3-OH), and iso-C_10:0_. PC, SGL, PE, PG, DPG are identified as major polar lipids. The DNA G+C content calculated from the whole genome sequence of the type strain is 57.5 mol%.

The type strain is DGS5-3^T^ (=KACC 23854^T^ =JCM 37497^T^), isolated from the marine red alga *Chondrus* sp. collected at Donggo-ri Beach, Republic of Korea. The GenBank accession numbers for the 16S rRNA gene and whole-genome sequences of strain DGS5-3^T^ are PQ270117 and CP170454, respectively.

### Emended description of the genus *Qipengyuania* Xu *et al*. 2020

The description is based on that provided by Xu *et al*. [2], with the following amendments. The DNA G+C content ranges from 56.5% to 73.7%, and the genome sizes range from 2.6 to 3.5 Mb, as determined from whole-genome sequences.

## Supplemental Materials

Supplementary data for this paper are available on-line only at http://jmb.or.kr.



## Figures and Tables

**Table 1:** General genomic features of strains DGS2-2^T^ and DGS5-3^T^ and closely related type strains of the genus *Qipengyuania*.

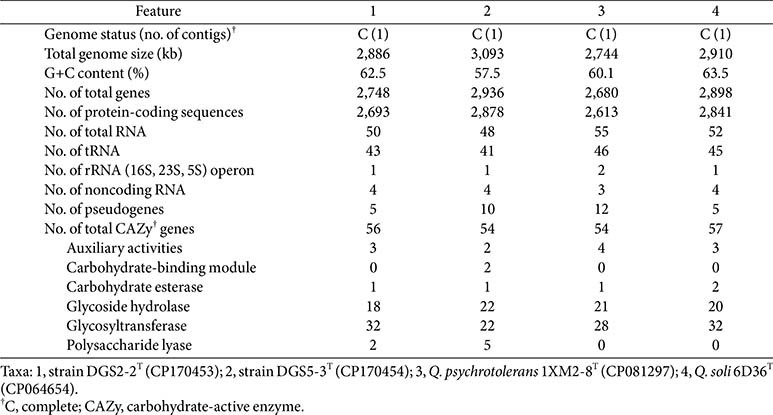

**Table 2:** Differential characteristics between strains DGS2-2^T^ and DGS5-3^T^ and closely related type strains of the genus *Qipengyuania*.