# Supplementary material for: Carotenoid-Producing Qipengyuania algicola sp. nov. and Qipengyuania rhodophyticola sp. nov., Isolated from Marine Algae, and Emended Description of the Genus Qipengyuania Xu et al. 2020
Source: J Microbiol Biotechnol. 2025 Sep 16;35:e2507023. doi: 10.4014/jmb.2507.07023 (PMC12476985; doi:10.4014/jmb.2507.07023)
Supplement: Supplementary file 1 [file jmb-35-e2507023-supple.pdf]

**J. Microbiol. Biotechnol. 2025. 35: e2507023**

https://doi.org/10.4014/jmb.2507.07023

# Supplementary Figures and Tables

## Carotenoid-producing *Qipengyuania algicola* sp. nov. and *Qipengyuania rhodophyticola* sp. nov., isolated from marine algae, and emended description of the genus *Qipengyuania* Xu *et al*. 2020

Jae Kyeong Lee†, Min Woo Lee†, Chae Yeong Moon, Jeong Min Kim, Hülya Bayburt, Byeong Jun Choi, and Che Ok Jeon*

*Department of Life Science, Chung-Ang University, Seoul 06974, Republic of Korea*

†These authors contributed equally to this work.

*Author for correspondence: Che Ok Jeon (cojeon@cau.ac.kr)

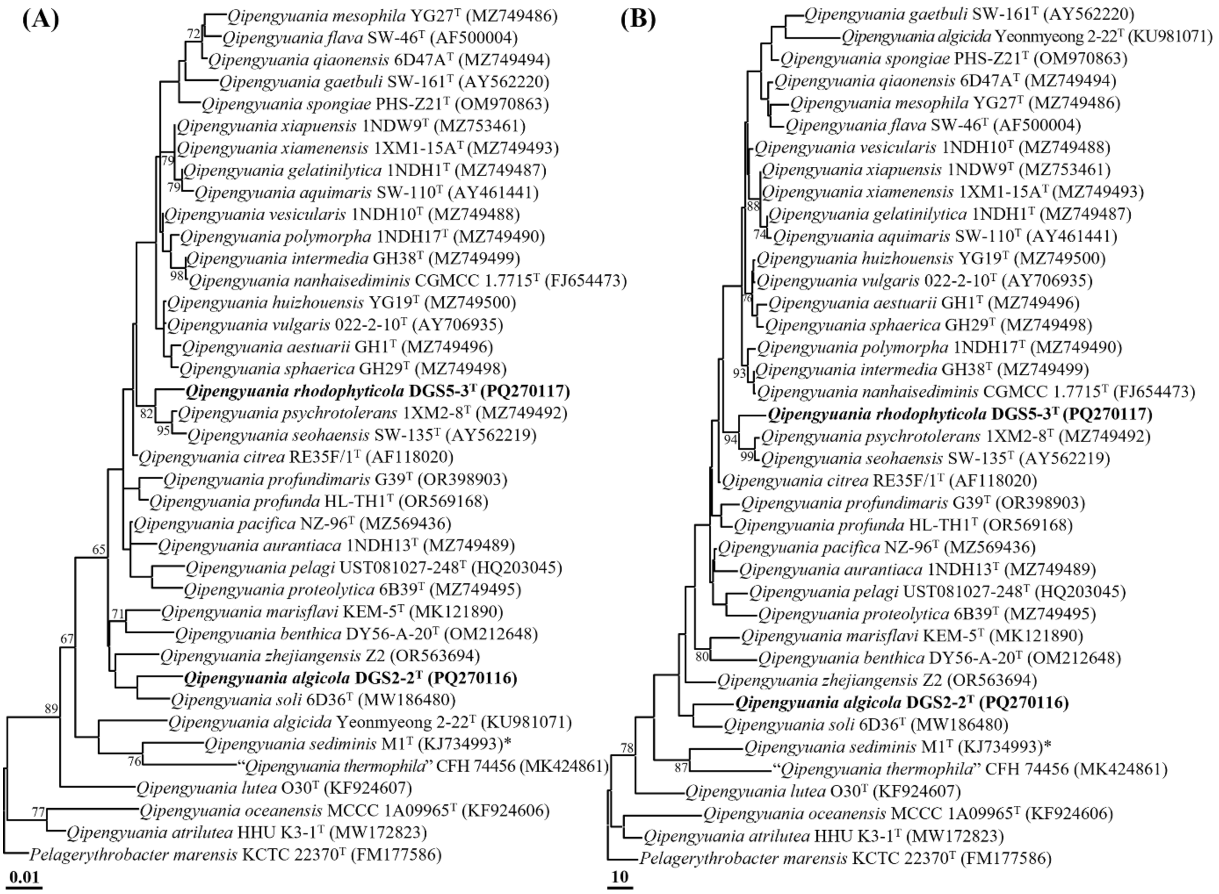


**Fig. S1. Maximum-likelihood (A) and maximum-parsimony (B) trees showing the phylogenetic relationships of strains DGS2-2$^{T}$ and DGS5-3$^{T}$ and their closely related taxa, based on 16S rRNA gene sequences.** Bootstrap values (>70%) from 1,000 replicates are shown at branch nodes. *Pelagerythrobacter marensis* DSM 21428$^{T}$ (FM177586) was employed as an outgroup, and the type species of the genus *Qipengyuania* is denoted with an asterisk (*). Scale bars in panels A and B represent substitutions per nucleotide and nucleotide substitutions over the entire sequences, respectively.

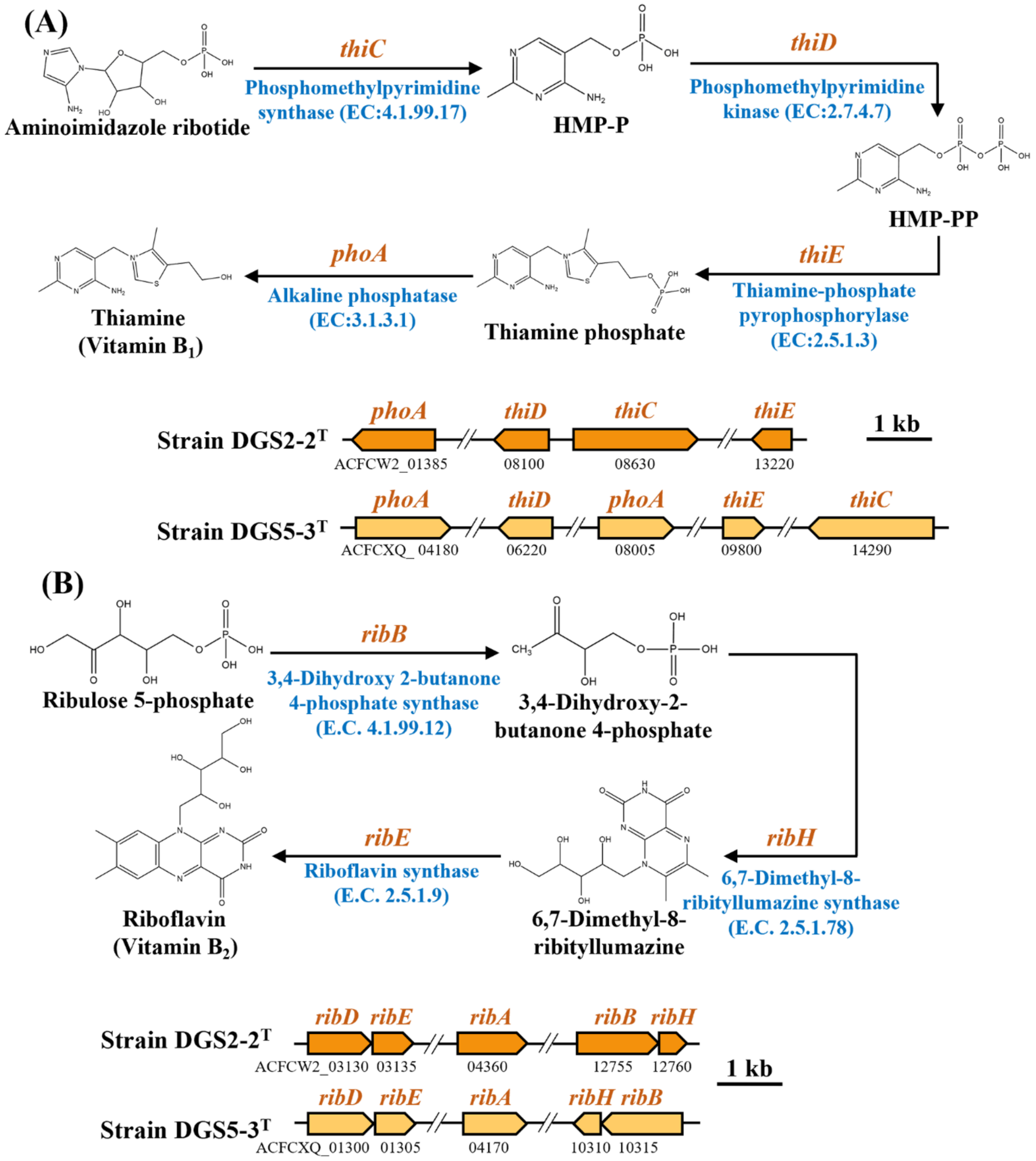


**Fig. S2. Metabolic pathways and genes involved in the biosynthesis of thiamine (vitamin $B_1$) from aminoimidazole ribotide (A)** and riboflavin (vitamin $B_2$) from ribulose-5-phosphate **(B)** identified in strains DGS2-2$^T$ and DGS5-3$^T$.

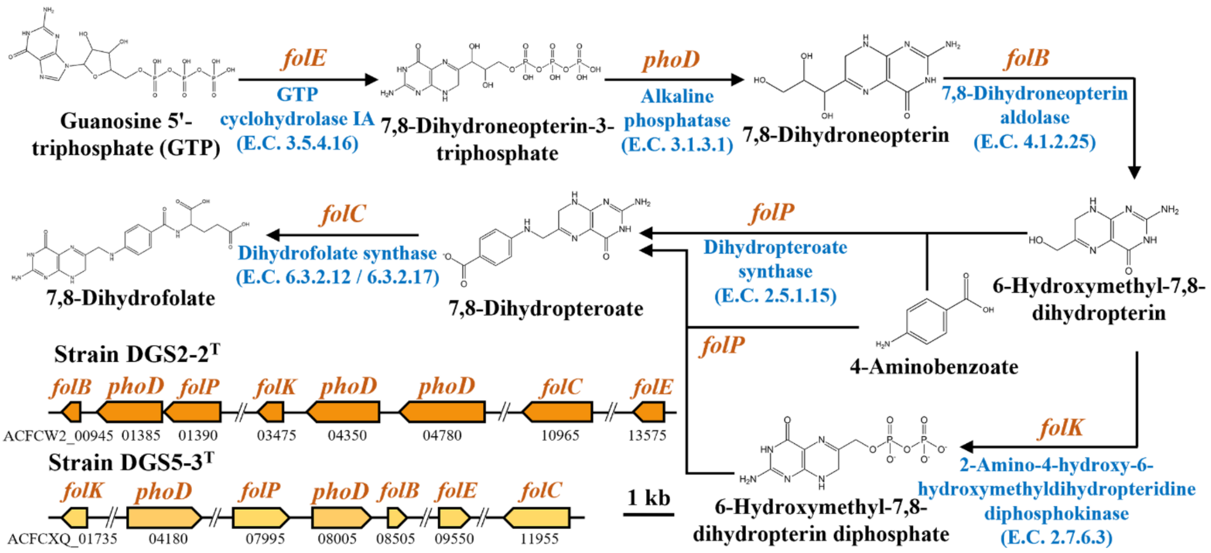


**Fig. S3.** Metabolic pathway and genes involved in the biosynthesis of 7,8-dihydrofolate, a precursor of folate (vitamin $B_9$), from GTP identified in strains DGS2-$2^T$ and DGS5-$3^T$.

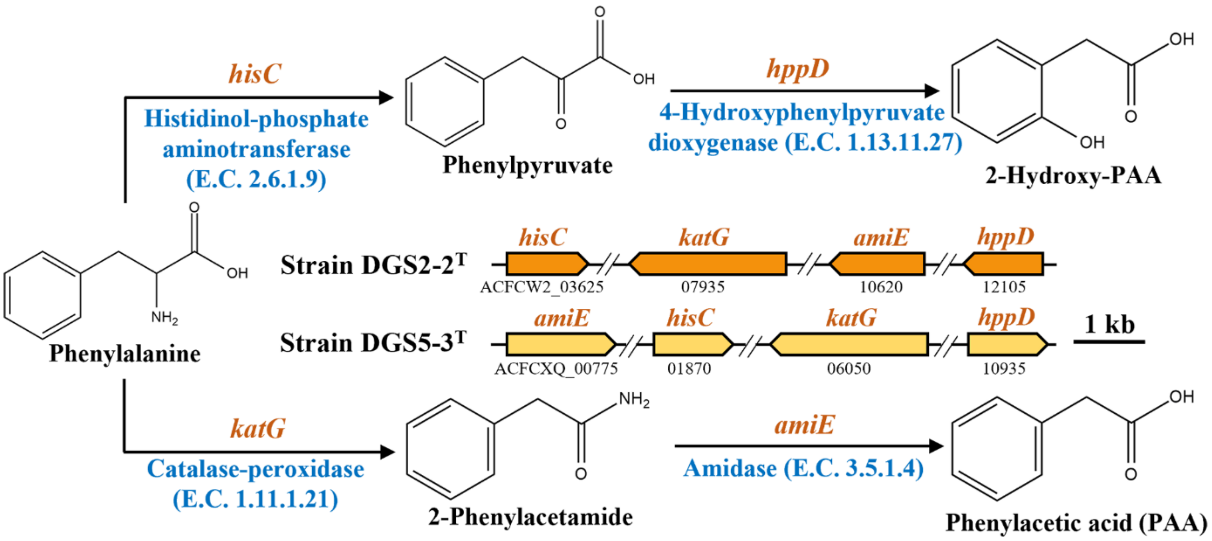


**Fig. S4. Metabolic pathways and genes involved in the biosynthesis of phenylacetic acid (PAA) and 2-hydroxy-PAA from phenylalanine identified in strains DGS2-$2^T$ and DGS5-$3^T$.**

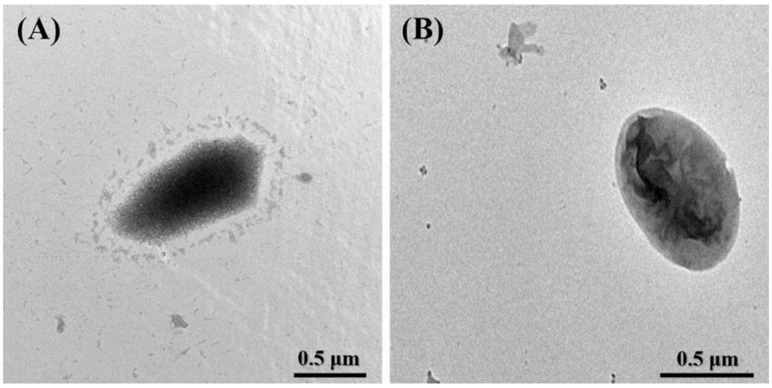


**Fig. S5. Transmission electron micrographs of negatively stained cells of strains DGS2-2ᵀ (A) and DGS5-3ᵀ (B), prepared with 2% (w/v) uranyl acetate, showing their general morphology after cultivation on marine agar at 30°C for 3 days.**

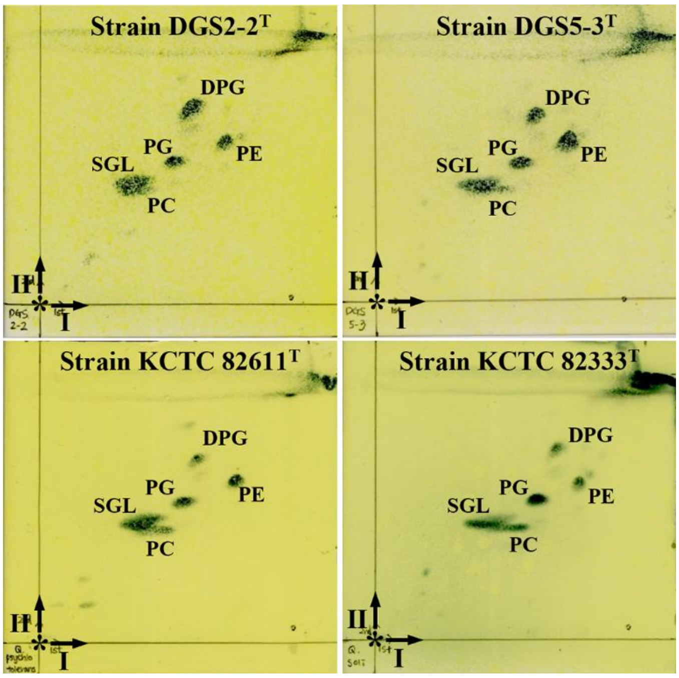


**Fig. S6. Two-dimensional thin-layer chromatograms (TLC) showing the total polar lipid profiles of strains DGS2-2ᵀ and DGS5-3ᵀ and their closely related *Qipengyuania* type strains, *Q. psychrotolerans* KCTC 82611ᵀ and *Q. soli* KCTC 82333ᵀ.** Solvent systems: (I) chloroform-methanol-water (65:25:4, v/v/v) and (II) chloroform-acetic acid-methanol-water (80:15:12:4, v/v/v/v). The TLC plates were sprayed with 10% ethanolic molybdophosphoric acid for the detection of total polar lipids. PC, phosphatidylcholine; SGL, sphingoglycolipid; PE, sphingoglycolipid; PG, phosphatidylglycerol; DPG, diphosphatidylglycerol.

**Table S1. Genome relatedness among strains DGS2-2$^T$ and DGS5-3$^T$ and closely related type strains of the genus *Qipengyuania*.**

| | | dDDH† value (%) | | | |
|---|---|---|---|---|---|
| | | **1** | **2** | **3** | **4** |
| ANI† value (%) | **1** | – | 18.4 | 19.5 | 19.4 |
| | **2** | 72.0 | – | 18.2 | 18.4 |
| | **3** | 72.6 | 71.6 | – | 19.0 |
| | **4** | 72.7 | 71.8 | 74.5 | – |

Taxa: 1, strain DGS2-2$^T$ (CP170453); 2, strain DGS5-3$^T$ (CP170454); 3, *Q. psychrotolerans* 1XM2-8$^T$ (CP081297); 4, *Q. soli* 6D36$^T$ (CP064654).

†ANI, average nucleotide identity; dDDH, digital DNA-DNA hybridization.

**Table S2. Cellular fatty acid compositions (%) of strains DGS2-2$^{T}$ and DGS5-3$^{T}$ and closely related type strains of the genus *Qipengyuania***

| Fatty acid | 1 | 2 | 3 | 4 |
|---|---|---|---|---|
| Saturated: | | | | |
| $C_{10:0}$ | 4.7 | **6.3** | 1.8 | 4.9 |
| $C_{12:0}$ | **5.1** | 4.7 | 1.2 | 1.5 |
| $C_{14:0}$ | 1.1 | 2.7 | tr | 1.1 |
| $C_{16:0}$ | **10.1** | **9.1** | **7.0** | **6.3** |
| $C_{17:0}$ | 3.5 | 2.9 | 3.1 | 2.5 |
| $C_{18:0}$ | 2.0 | **6.0** | tr | 1.1 |
| Unsaturated: | | | | |
| $C_{17:1}$ *ω6c* | **11.9** | 1.9 | **22.1** | **19.1** |
| $C_{17:1}$ *ω8c* | 1.9 | 3.1 | 1.7 | 1.8 |
| iso-$C_{17:1}$ *ω5c* | – | 2.6 | – | – |
| $C_{18:1}$ *ω7c* 11-methyl | 3.7 | – | 4.3 | **5.5** |
| Branched: | | | | |
| iso-$C_{10:0}$ | – | **5.2** | – | – |
| iso-$C_{12:0}$ | – | 2.2 | tr | – |
| iso-$C_{17:0}$ | tr | 1.7 | tr | – |
| iso-$C_{19:0}$ | – | 2.6 | – | tr |
| anteiso-$C_{17:1}$ | – | 2.5 | tr | – |
| anteiso-$C_{17:1}$ A | 1.7 | – | – | – |
| Hydroxy: | | | | |
| $C_{10:0}$ 3-OH | **5.4** | – | – | – |
| $C_{11:0}$ 2-OH | – | 2.1 | – | 2.7 |
| $C_{12:0}$ 2-OH | 1.8 | – | – | – |
| $C_{14:0}$ 2-OH | tr | 1.5 | 3.7 | 2.2 |
| $C_{15:0}$ 2-OH | 2.2 | 2.9 | **6.2** | **5.3** |
| $C_{16:0}$ 2-OH | 1.3 | – | 1.4 | 2.0 |
| iso-$C_{11:0}$ 3-OH | – | 1.5 | tr | – |
| iso-$C_{12:0}$ 3-OH | 1.1 | 4.0 | tr | tr |
| iso-$C_{15:0}$ 3-OH | – | – | 1.1 | tr |
| iso-$C_{17:0}$ 3-OH | 1.5 | – | – | – |
| Summed feature*: | | | | |
| 1 | 1.6 | **5.4** | 1.1 | 1.6 |
| 3 | 4.1 | **10.1** | **7.3** | **8.8** |
| 6 | – | 3.5 | – | – |
| 8 | **28.0** | **13.0** | **27.0** | **20.3** |
| 9 | – | – | 1.4 | 2.3 |

Taxa: 1, strain DGS2-2$^{T}$; 2, strain DGS5-3$^{T}$; 3, *Q. psychrotolerans* KCTC 82611$^{T}$; 4, *Q. soli* KCTC 82333$^{T}$. All data were obtained from this study. Data are expressed as percentages of the total fatty acids, and fatty acids constituting less than 1.0% in all strains are not shown. Major components (>5.0%) are highlighted in bold. Symbols: tr, trace amount (<1.0%); –, not detected.

*Summed Features are fatty acids that cannot be resolved reliably from another fatty acid using the chromatographic conditions chosen. The MIDI system groups these fatty acids together as one feature with a single percentage of the total. Summed features 1, iso-$C_{15:1}$ H and/or $C_{13:0}$ 3-OH; 3, $C_{16:1}$ *ω7c* and/or $C_{16:1}$ *ω6c*; 6, $C_{19:1}$ *ω11c* and/or $C_{19:1}$ *ω9c*; 8, $C_{18:1}$ *ω7c* and/or $C_{18:1}$ *ω6c*; 9, iso-$C_{17:1}$ *ω9c* and/or 10-methyl $C_{16:0}$.
